# Timing the tides: Genetic control of diurnal and lunar emergence times is correlated in the marine midge *Clunio marinus*

**DOI:** 10.1186/1471-2156-12-49

**Published:** 2011-05-20

**Authors:** Tobias S Kaiser, Dietrich Neumann, David G Heckel

**Affiliations:** 1 Department of Entomology, Max Planck Institute for Chemical Ecology, 07745 Jena, Germany; 2 Max F. Perutz Laboratories, Dr. Bohr-Gasse 9, 1030 Wien, Austria; 3 Institute of Zoology, University of Cologne, Biozentrum, Zülpicher Straße 47-B, 50674 Köln, Germany

## Abstract

**Background:**

The intertidal zone of seacoasts, being affected by the superimposed tidal, diurnal and lunar cycles, is temporally the most complex environment on earth. Many marine organisms exhibit lunar rhythms in reproductive behaviour and some show experimental evidence of endogenous control by a circalunar clock, the molecular and genetic basis of which is unexplored. We examined the genetic control of lunar and diurnal rhythmicity in the marine midge *Clunio marinus *(Chironomidae, Diptera), a species for which the correct timing of adult emergence is critical in natural populations.

**Results:**

We crossed two strains of *Clunio marinus *that differ in the timing of the diurnal and lunar rhythms of emergence. The phenotype distribution of the segregating backcross progeny indicates polygenic control of the lunar emergence rhythm. Diurnal timing of emergence is also under genetic control, and is influenced by two unlinked genes with major effects. Furthermore, the lunar and diurnal timing of emergence is correlated in the backcross generation. We show that both the lunar emergence time and its correlation to the diurnal emergence time are adaptive for the species in its natural environment.

**Conclusions:**

The correlation implies that the unlinked genes affecting lunar timing and the two unlinked genes affecting diurnal timing could be the same, providing an unexpectedly close interaction of the two clocks. Alternatively, the genes could be genetically linked in a two-by-two fashion, suggesting that evolution has shaped the genetic architecture to stabilize adaptive combinations of lunar and diurnal emergence times by tightening linkage. Our results, the first on genetic control of lunar rhythms, offer a new perspective to explore their molecular clockwork.

## Background

Lunar rhythms of reproduction are found in a number of species, especially from the intertidal zone [1-7], where the recurrent alternation between marine and terrestrial conditions results in huge fluctuations in abiotic factors. Certain tidal conditions re-occur predictably during the lunar cycle and the adaptive value of lunar rhythms can likely be attributed to restricting the delicate events of reproduction to suitable, narrow windows of time [7,8]. Lunar rhythms in reproduction are also reported for other marine species that do not experience tidal fluctuations, one of the most famous examples being the swarming of the Palolo Worm *Eunice viridis *in Samoa [9,10]. They are also reported for plankton and insects of tropical lakes [11,12]. In these cases the likely adaptive value of the lunar rhythm is to synchronize reproduction within populations in the absence of distinct seasonality. In both contexts, slight differences in the reproductive rhythms can easily lead to reproductive isolation between populations [13-15].

Despite the widespread occurrence of lunar rhythms and their impact on the development and reproduction of marine species, their physiological and molecular basis is largely unknown. The first important question following the discovery of a lunar rhythm is whether it is controlled by a biological clock and therefore endogenous, or whether it is merely a direct response to external cues in the lunar cycle such as the increased illumination during full moon nights. The critical evidence for an endogenous rhythm is that the rhythm must *free-run*: After being *entrained *by a specific environmental cue, the so-called *zeitgeber*, the rhythm must continue under constant conditions, in the absence of that particular cue. This has only been shown for the lunar rhythms of a few species [1-4,16-21]. Such proof is lacking for the coral *Acropora millepora *and the fish *Siganus guttatus*, so that their recently-documented transcriptional differences in relation to the lunar cycle [22,23], which represent the only published molecular studies on lunar rhythms to date, cannot necessarily be attributed to a biological clock.

One of the most striking and best studied endogenous lunar rhythms is found in the marine midge *Clunio marinus *HALIDAY 1855 (Diptera, Chironomidae) [24]. This species occupies the intertidal zone of rocky shores along the European Atlantic coast. While the larvae need to be constantly submerged, and thus settle at the lower fringe of the eulittoral, the adults need the larval substrates to be exposed for oviposition. The evolutionary solution to this conflict has been to drastically reduce adult lifespan to only a few hours, and to fine-tune adult emergence exactly to the time when the water is as low as possible. This occurs during the spring tides (around new moon and full moon) shortly before the time of low tide. Thus, adult emergence in *Clunio *is characterized by semilunar (every spring tide) or lunar (every second spring tide) rhythms as well as a diurnal rhythm. Upon emergence the midges immediately mate and oviposit and then die in the rising tide.

The days of spring tide and the corresponding lunar emergence days of different *Clunio *populations are similar for all places along the coast [3]. However, the time of low tide on spring tide days varies tremendously among different localities, and the diurnal emergence times of *Clunio *populations in the field have been found to be locally adapted to the respective time of low tide [3,25]. Both (semi)lunar and diurnal emergence have been shown in laboratory experiments to be controlled by biological clocks [3]. The light-dark (LD) cycle has been shown to act as a zeitgeber for the circadian rhythm [3], while for the circa(semi)lunar rhythm three cues were shown to be effective: moonlight, tidal fluctuations in water turbulence, and temperature [3,25,26].

We investigated the genetic basis of the diurnal and lunar rhythms of *Clunio marinus *by crossing strains collected from two populations differing in diurnal and lunar time of emergence and in the period of the lunar rhythm, i.e. the time between consecutive emergence peaks. These differences have been maintained in the laboratory for more than three years under artificial LD and lunar entrainment. We found that the differences in both lunar and diurnal emergence time are under genetic control, and there is a genetic basis for correlation between the two timing phenotypes that is adaptive in the natural environment.

## Results

### Genetic control of the diurnal emergence rhythm

**Figure 1:**
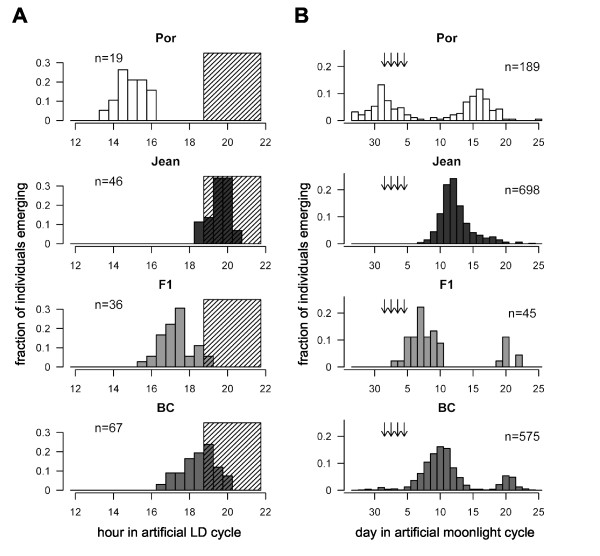
**Emergence patterns of parental strains, hybrids, and backcrosses of two populations of *Clunio marinus***. (A) Diurnal rhythm plotted as the fraction of individuals that emerged during 30 minute intervals. Parental strain of Port-en-Bessin (n = 19). Parental strain of St. Jean-de-Luz (n = 46). F1 generation (n = 36). Backcross generation (n = 67). Daytime is given in hours after the middle of dark phase ("hour 0"), which necessarily makes the middle of the light phase "hour 12". The grey shading marks dark phase. For the backcross only the single reared crosses were recorded in 30 min intervals and are given here. Comprehensive diurnal data is available in [3]. (B) Lunar rhythm plotted as the fraction of individuals that emerged during each day of the artificial moonlight cycle. Parental strain of Port-en-Bessin (n = 189). Parental strain of St. Jean-de-Luz (n = 698). F1 generation (n = 45). Backcross generation (n = 575). Arrows mark the days with artificial moonlight. Data of two lunar cycles added up.

Previous results with mass crosses using laboratory strains collected from the French Atlantic coast at Port-en-Bessin (Normandie; referred to as Por strain) and St. Jean-de-Luz (Basque Coast; referred to as Jean strain) suggested polygenic control of diurnal emergence time, as the continuously-distributed progeny emergence times of F1, F2 and backcross (BC) to Por occurred intermediately between their respective parents [27]. To further characterize these genetic factors, we conducted single pair intercrosses and backcrosses (to Jean) with strains collected from the same locations 40 years later, which were found to still maintain the previously-observed timing differences when reared under the same diurnal and lunar entrainment regimes in the laboratory (Table 1). As before, F1 adults emerged at times intermediate to the parental strains, and the adults formed by backcrossing the F1 to the Jean strain emerged between these two (Figure 1A, Table 1). Testing the form of this observed backcross distribution against expectations based on action of 1, 2, or more unlinked genes significantly rejected the single-factor hypothesis (Table 2). The earlier data based on mass crosses fit the two-factor model best (Table 2). Thus the simplest hypothesis is that two major genetic factors are involved in the stable maintenance of differences in diurnal emergence time among these two populations.

**Table 1:** Observed and expected values of the timing traits for parents, F1 and BC.

	Diurnal rhythm	Lunar rhythm	
		
	Time in LD cycle (hour)	Time in artificial moonlight cycle (day)	
		Peak 1	Peak 2
St. Jean-de-Luz	19.8 (0.6)	12.4 (2.4)	-
Port-en-Bessin	15.2 (0.7)	1.2 (2.3)	15.6 (2.3)
			
F1 expected	17.5	6.8	
F1 actual	17.6 (0.8)	7.2 (1.8)	20.4 (1.1)
			
BC expected	18.7	9.8	
BC actual	18.7 (0.9)	9.7 (2.4)	20.5 (1.1)

Values in brackets are standard deviations.

**Table 2:** Statistics of the deviation of the observed BC distributions of diurnal and lunar emergence times from expected distributions based on genetic models for different numbers of genes.

a) Kolmogorov-Smirnov test for goodness of fit.						
**Nr of genes**	**Diurnal rhythm**				**Lunar rhythm**	
		
	**BC** **n = 62**		**BC (Neumann 1967)** **n = 816**		**BC** **n = 487**	
			
	**D**	**D**_**0.05**_	**D**	**D**_**0.05**_	**D**	**D**_**0.05**_

1	**0.18**	**0.17**	**0.135**	**0.048**	**0.16**	**0.06**
2	0.04	0.17	0.048	0.048	0.04	0.06
3	0.06	0.17	**0.079**	**0.048**	0.05	0.06
4	0.14	0.17	**0.087**	**0.048**	0.03	0.06
5	0.09	0.17	**0.080**	**0.048**	0.06	0.06
6	0.10	0.17	**0.107**	**0.048**	0.05	0.06

If D is larger than D_0.05_, the hypothesis is rejected at the 5% level.						
Genetic models which are rejected are given in bold numbers.						
b) Chi-Square test for independence (G-test)						

**Nr of genes**	**Diurnal rhythm**				**Lunar rhythm**	
		
	**BC** **n = 62**		**BC (Neumann 1967)** **n = 816**		**BC** **n = 487**	
			
	**G**	**P**	**G**	**P**	**G**	**P**

1	**25.66**	**0.004**	**80.53**	8.7*10^**-12**^	**97.90**	5.4*10^**-13**^
2	5.54	0.85	**26.12**	**0.02**	22.58	0.21
3	3.70	0.96	**39.61**	**0.0002**	15.91	0.60
4	4.33	0.93	**63.43**	1.3*10^**-8**^	12.08	0.84
5	6.18	0.80	**79.50**	1.4*10^**-11**^	22.33	0.22
6	10.71	0.38	**147.16**	7.7*10^**-25**^	22.00	0.23

### Genetic control of the lunar emergence rhythm

The same single pair crosses and additional mass reared crosses also revealed a complex genetic pattern of lunar emergence dates (Figure 1B, Table 1). The lunar zeitgeber consisted of artificial moonlight presented during the first 4 nights of a 30-day cycle. Since larval growth rates in *Clunio *differ greatly even within families, the individuals of one generation typically emerge in several cycles over 2 to 3 months. Jean strain individuals emerged in a lunar rhythm, within a single peak in a 30-day cycle at around day 12. The Por strain showed a semilunar rhythm, with emergences distributed equally into two peaks in a 30-day cycle centred at days 1 and 16. Thus the parental strains differed both in the period of the rhythm and the timing of the peaks, and these two properties behaved differently in the crosses.

Most progeny emerged in a single peak, its phasing and breadth differing for F1 and BC generations. The major peak in the F1 (7.2) is intermediate between Jean (12.4) and the first peak in Por (1.2) (Table 1). This suggests that the first Por peak (and not the second) corresponds physiologically to the Jean peak, and also to the major F1 peak. The major BC peak contains progeny with major F1 peak-phasing and progeny with Jean peak-phasing. Segregation of a single gene determining major peak-phasing would predict that these are the only two classes of progeny in the major BC peak, present in equal frequency. However, analysis of the phenotype distribution rejects this hypothesis; the trait is polygenic. It is not possible to give a precise estimate of the number of genes, as the phenotype distributions of the F1 and the Jean strain overlap too much.

A fraction of the progeny emerged at times outside the major peak; 18% of the F1, and 15% of the BC. This may reflect a genetic influence of the Por parent which shows two peaks of emergence in a semi-lunar rhythm; alternatively it could represent an irregular response of some individuals to the artificial moonlight entrainment regime, as has been observed previously in the Jean strain [3]. As it is not clear if these minor peaks have a genetic basis, the experiment remains inconclusive with respect to the inheritance of the period of the lunar rhythm. But notably, in the BC the major peak is spread due to the segregation of F1 and Jean phenotypes and slightly skewed towards the Jean phenotypes (Figure 1 B). At the same time the minor BC peak is lacking the segregational spread and is F1-like in timing. Both phenomena could theoretically be explained by genetic linkage of a gene allowing emergence in the minor peak (and coming from the Por parent) to one of the genes controlling lunar emergence time.

### Correlation of diurnal and lunar emergence times

**Figure 2:**
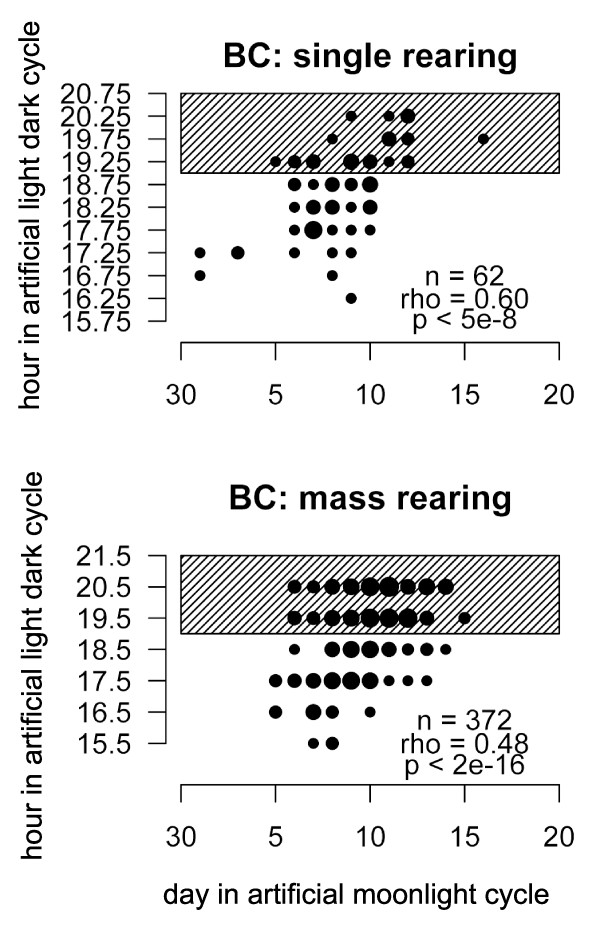
**Correlation of lunar and diurnal emergence times for the major peak of the BC progeny**. Hour of diurnal emergence is plotted against day of the artificial moonlight cycle. Circle area is proportional to the number of individuals at the respective time point. The shaded area marks dark phase. (A) Single rearing. (B) Mass rearing. There were six additional individuals emerging on days 1 to 4 for which diurnal emergence time is not known; there were no more individuals emerging after day 15.

If lunar emergence dates and diurnal emergence times were inherited independently, random combinations of both traits would be expected among the backcross progeny. However, the test for correlation of the two traits in the BC is highly significant in both the single crosses, for which diurnal emergence times were recorded in half-hour intervals, and the mass reared BC individuals, for which diurnal emergence times were recorded in hourly intervals (Figure 2). Since there was no correlation in the F1 (ρ = -0.12, P < 0.55), it is not likely that the BC correlation is brought about by external factors. Based on the single crosses, which are genetically less heterogeneous and have the more precise diurnal data, we tested the level of genetic linkage required to achieve a correlation of the given magnitude, for 2-4 genes controlling diurnal and 2-4 genes controlling lunar emergence time. We found that only very high levels of linkage can explain the correlation (Table 3). The simplest model that fits the data assumes that each of the timing traits is determined by two independent, unlinked genes and that one of the genes controlling diurnal emergence time is tightly linked to (or the same as) one of the genes controlling lunar emergence time, while the other gene controlling diurnal emergence time is tightly linked to (or the same as) the other gene controlling lunar emergence time. However, more complex models, involving more factors with varying degrees of partial linkage, cannot be ruled out.

**Table 3:** Probability (P) values for models of genetic linkage.

		Lunar genes											
		
		2			3				4				
Linkages		0	1	2	0	1	2	3	0	1	2	3	4

Circadian genes	2	< 10^-6^	0.0002	0.453	n.t.	n.t.	0.053	-	n.t.	n.t.	n.t.	-	-
	3	n.t.	n.t.	0.047	n.t.	n.t.	0.003	0.404	n.t.	n.t.	n.t.	0.095	-
	4	n.t.	n.t.	n.t.	n.t.	n.t.	n.t.	0.092	n.t.	n.t.	n.t.	0.015	0.382

Probability (P) values are based on the quality of the correlation achieved by a given genetic model in 100,000 replications as compared to the observed BC. Underlined values indicate models that are not rejected at the 0.05 level.

n.t. = not tested (see Materials and Methods)

## Discussion

### Local adaptation in lunar emergence time

**Figure 3:**
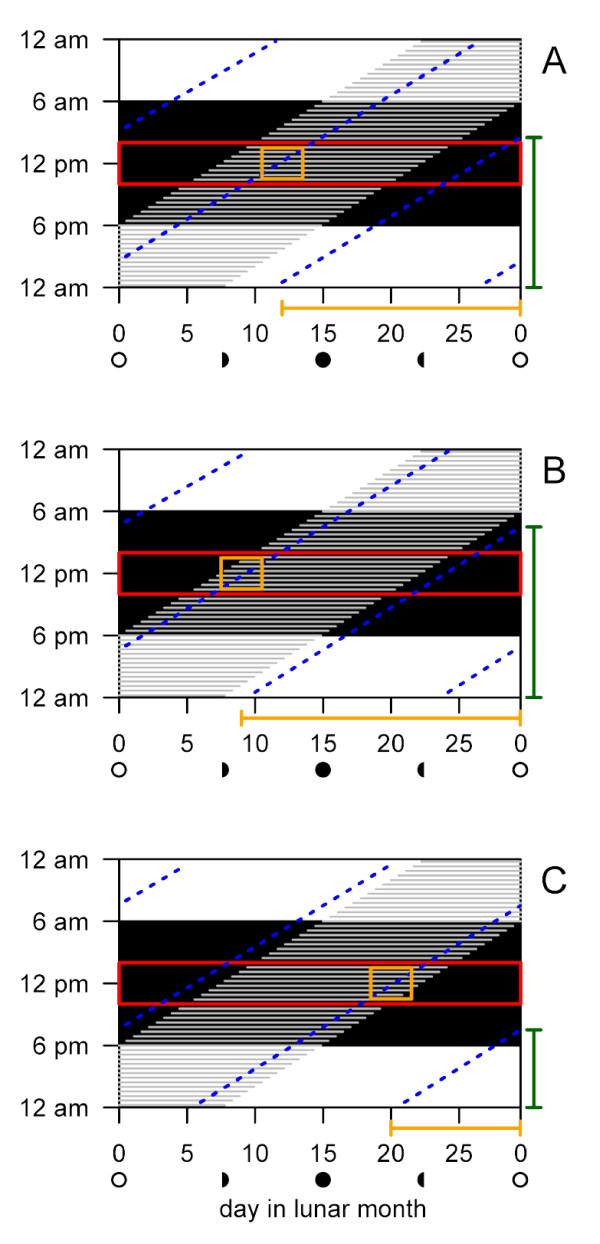
**Schematic relationship of the moon phases, the tides, *Clunio's *moonlight sensitivity and the locally adapted diurnal and lunar emergence times**. Time of day (in hours) is plotted against time in the lunar cycle (in days). The black area represents the dark phase. The grey shading indicates when the moon is in the sky. The red box marks the circadian period of sensitivity of *Clunio *to moonlight [24,28]. As a consequence, moonlight can theoretically be detected throughout the moonlit quarters around full moon. We hypothesize that the water level additionally influences the detectability of moonlight: Moonlight is best perceived when the time of low tide (blue dotted lines) falls to midnight, so that presence of the moon in the sky, *Clunio's *moonlight sensitivity and the low tide coincide (yellow box). As tidal regimes differ for other places along the coast (compare A, B and C), the time when moonlight is best perceived differs. Nevertheless, all known *Clunio *populations emerge during the spring tides, i.e. short after new moon and/or full moon. Thus, they must respond to the moonlight stimulus with a different delay of their emergence peak (indicated by the yellow bars below the graph). According to our hypothesis this should correspond to the time between the artificial moonlight treatment and the emergence peak in the respective laboratory strain (see Figure 4). Note, that the time span between low tide at midnight and full moon/new moon (yellow bars) is highly correlated with the daytime of low tide on full moon/new moon days (green bars, compare Figure 5).

**Figure 4:**
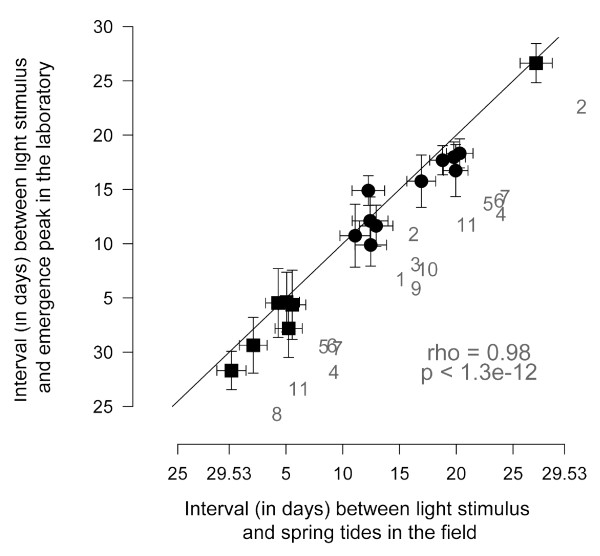
**Lunar emergence times of *Clunio *laboratory strains are adapted to the tidal regime at their place of origin**. We define the event of moonlight stimulus perception as the day when a low tide falls between 11 p.m. and 1 a.m. during the moonlit quarters of the lunar cycle. For different places along the coast, this event occurs at different phases of the lunar cycle, depending on the local tidal regime (compare Figure 3). The interval (in days) from this event to the spring tides in the field is plotted on the X-axis for each population. The interval (in days) between onset of artificial moonlight and the emergence peak in the laboratory is plotted on the Y-axis for the same populations. Error bars are standard deviations. Squares mark emergence peaks that fall to full moon, circles mark emergence peaks that fall to new moon in the field. Correlation coefficient and p value of the correlation are given in the graph. Strain identities: 1 Vigo (West Spain). 2 Santander (North Spain). 3 St. Jean-de-Luz (Basque Coast, France). 4 Port-en-Bessin (Normandie, France). 5 Lulworth (English Channel, UK). 6 Studland (English Channel, UK). 7 Bembridge (Isle of Wight, UK). 8 Roscoff (Bretagne, France). 9 Concarneau (Bretagne, France). 10 Camaret-sur-Mer (Bretagne, France). 11 St. Briac-sur-Mer (Bretagne, France). Data from [3,30,53]. For strains 9 to 11 own unpublished data is given.

The artificial moonlight stimulus, which is widely used when studying lunar rhythms in the laboratory, is generally assumed to correspond to natural full moon in the field. However, the different, genetically determined time intervals between the artificial moonlight stimulus and the emergence peaks in the laboratory (Figure 1B) do not conform to the observation that the corresponding field populations of *Clunio marinus *all emerge around full moon and new moon in the field, i.e. in the same relation to the natural full moon. This questions the equivalence of laboratory artificial moonlight to the natural full moon. In fact, the visibility of moonlight to *Clunio *larvae in submerged substrates in the field could depend on the phase of the moon as well as water levels [28]. Intertidal *Clunio *larvae are only sensitive to moonlight around midnight [24,28], and thus moonlight can probably only act as a zeitgeber when there is a low tide around midnight. Since tidal regimes differ, this happens at different times of the lunar cycle in different places (Figure 3). Therefore, we compared the time interval between artificial moonlight and the emergence peak of laboratory strains with the time interval between low-tide at midnight and the spring tide days at their place of origin (Figure 4). The good correlation suggests that indeed the differing reactions of different populations to laboratory artificial moonlight reflect a genetic adaptation of the lunar emergence rhythm of those populations to the local tidal regime in the field. In addition to the known local adaptations in diurnal emergence time, this adds a novel, second level of local adaptation to the complex timing traits in *Clunio marinus*. At the same time, it argues for caution when interpreting the biological meaning of the artificial moonlight stimulus for other species.

### What is the basis of the correlation of diurnal and lunar emergence times?

The explanation for the genetic non-independence of lunar and diurnal emergence times can range from genetic linkage of the timing genes in a one-by-one fashion, to involvement of the same genes in both processes. Regarding the latter scenario we can further distinguish three cases: identity of the lunar and the circadian clockworks; modular pleiotropy [29], i.e. an influence of the circadian clock as a whole on lunar timing (or vice versa); and gene pleiotropy, i.e. the shared use of single genes in both processes.

Identity of the lunar and the circadian clocks is difficult to imagine and highly unlikely, given the completely different properties of these clocks, e.g. the different periods, the different zeitgebers and the different responses to entrainment. In *Clunio*, the circadian oscillator, i.e. the cycling process at the core of the circadian clock, responds to phase-shifts, i.e. changes in the cycle of the environmental zeitgeber, with transient cycles. In contrast, the lunar oscillator is reset immediately [Neumann, unpublished].

Modular pleiotropy is much easier to conceive, especially as the circadian clock is known to play a role in regulating moonlight sensitivity in *Clunio *[24,30]. For example, the differing diurnal emergence peaks of the Jean and Por strains (Figure 1A) could be due to their circadian oscillators differing in phase by 4.6 hours, i.e. because they are cycling at a 4.6 hour offset. This could affect lunar timing by causing the windows of nocturnal moonlight sensitivity to differ. However, this cannot account for the 11-day difference in lunar emergence between the two strains (Figure 1B). Artificial moonlight was presented throughout the night and should have been perceived as a stimulus no matter when the window of sensitivity occurred. Moreover, measurement of so-called phase response curves (PRCs) [31] has shown that the phase difference of the circadian oscillators of the two strains is much less than 4.6 hours and thus cannot even fully account for the observed difference in diurnal emergence peaks (Figure 1A). A similar situation was observed in selection experiments with *Drosophila pseudoobscura *which produced early and late emerging strains differing by 4 hours in diurnal emergence time but 0 hours in the phase of their circadian oscillators, as assayed by PRCs [32]. Therefore, the genetic components controlling diurnal emergence time are likely to act not within, but downstream of the circadian oscillator, as are any physiological interactions implied by the existence of two timing genes affecting both diurnal and lunar rhythms. This makes it unlikely that modular pleiotropy could account for the correlation of diurnal and lunar emergence times in *Clunio*.

A similar situation may occur with the photoperiodic response of diapause timing in the pitcher plant mosquito *Wyeomyia smithii*. Induction of diapause depends on a critical day length, which varies with latitude. The comparison between lunar periodicity and photoperiodic diapause induction is particularly interesting, because lunar rhythmicity has been suggested to be evolutionarily related to photoperiodic diapause induction [33]. Lunar zeitgeber perception in the former [28,34] and clock models of the latter [35,36] both have circadian regulation of photosensitivity at their basis, and both entail hormonal control of a developmental arrest. In *Wyeomyia smithii *photoperiodic adaptations in diapause along a south to north cline are not correlated to changes in the circadian clock [37]. This does not exclude a circadian regulation of photosensitivity as a basis to photoperiodism, but it parallels the finding that in *Clunio *the genetic factors for local adaptation are probably not part of the core circadian clock.

Gene pleiotropy might be more likely as the basis of the correlation between lunar and diurnal emergence times, and there is an example from the field of photoperiodism that illustrates this possibility. The *Drosophila melanogaster *clock gene *timeless (tim) *has a natural *ls-tim *variant that increases incidence of photoperiodic diapause independent of daylength [38]. This prompted the suggestion that it does not exert this effect via its role in the circadian clock [29], although *ls-tim *also affects light sensitivity of the circadian clock [39]. Other cases of gene pleiotropy from chronobiology are the *D. melanogaster glycogen synthase kinase 3 *(*gsk-3*, alias *shaggy*) and *casein kinase 2 *(*ck2*), which besides their role in the circadian clock [40,41] have documented roles in DNA repair (*ck2*) [42], microtubule assembly (*gsk-3*) [43] and wnt/wingless signalling (both) [44,45]. However, we have no candidate genes at hand with which to evaluate the plausibility of gene pleiotropy.

Thus when trying to explain the genetic correlation in terms of the same genes affecting both timing processes, three difficulties arise: identity of the molecular clockworks is highly unlikely, there is currently no support for an effect of modular pleiotropy in *Clunio*, and gene pleiotropy is possible but suffers from a lack of candidate genes. Thus an alternative hypothesis should be considered: that different genes affect circadian vs. lunar timing, and that lunar and circadian genes occur in linked pairs. While increasing the number of hypothesized genes, it removes the onus of explaining how one gene affects two timing mechanisms that appear physiologically distinct. Moreover, it is consistent with the existence of large non-pairing regions in two of the three *Clunio *polytene chromosomes, which likely are not subject to recombination [46].

### Evolutionary implications of the correlation for *Clunio*

**Figure 5:**
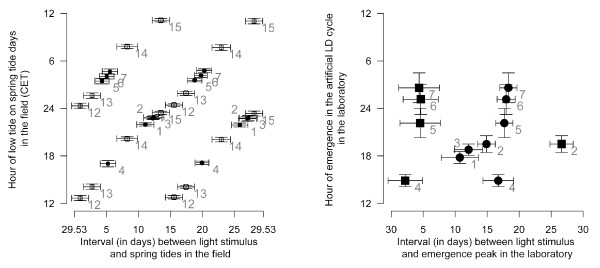
**Correlation of zeitgebers for different geographic locations and the corresponding adaptive combinations of lunar and diurnal emergence times.** (A) Correlation of zeitgebers in different geographic locations for full moon spring tides (squares) and new moon spring tides (circles). The laboratory strains' places of origin (filled squares and circles) and other reference localities (open squares and circles) are given with standard deviations. For the places of origin of the laboratory strains, only the low tide during which *Clunio *is known to emerge in the field is given, for the other reference places both daily low tides are given. (B) Combinations of lunar and diurnal emergence times in laboratory strains of different geographic origins. Peaks predicted to fall onto full moon are given as squares, those predicted to fall onto new moon are given as circles. The strains of Vigo (1) and St. Jean-de-Luz (3) have a lunar rhythm and do not emerge during full moon. Error bars are standard deviations. Strain identities: 1 Vigo (West Spain). 2 Santander (North Spain). 3 St. Jean-de-Luz (Basque Coast, France). 4 Port-en-Bessin (Normandie, France). 5 Lulworth (English Channel, UK). 6 Studland (English Channel, UK). 7 Bembridge (Isle of Wight, UK). Other reference places: 9 Devonport (English Channel, UK). 10 Ullapool (Scotland, UK). 11 Bremerhaven (Germany). 12 Brest (Bretagne, France). Data from [3,53]

Irrespective of the details of the genetic mechanism underlying the correlation of diurnal and lunar emergence times, the non-independence of the timing traits stabilizes well-adapted combinations of lunar and diurnal timing. The two phenomena to which lunar and diurnal emergence times are adapted, i.e. the number of days between the occurrence of low tide at midnight and the spring tide, and the time of low tide on spring tide days respectively, both depend on the local tidal regime (see Figure 3). Therefore, they are highly correlated at different locations along the coast (Figure 3, Figure 5A). The observed lunar and diurnal emergence times of different strains in the laboratory (Figure 5B) display adaptation to the tidal regime by mirroring the correlation in their original physical environments. This correlation in the timing phenotypes has a genetic basis, at least for the Por and Jean strains studied here; additional work will be required to establish whether the adaptive timing of strains from other localities is controlled by the same genetic factors and is genetically correlated in the same manner. This will elucidate whether the remarkable synchronization of diurnal and lunar rhythms used by *Clunio marinus *to time the tides emerges from a physiological constraint due to the involvement of the same genes in both, or whether the high predictability of the combinations of physical factors imposing selection has shaped genome architecture resulting in genetic linkage and stable combinations of the timing traits.

## Conclusion

As shown by crossing experiments, the diurnal and lunar emergence times in the marine midge *Clunio marinus *are both under genetic control. These two rhythms are correlated in a manner that facilitates local adaptation to tidal regimes that vary significantly among geographic locations. This is the first report on the genetic basis of variation in lunar rhythms in any species, and presents a totally unexpected genetic interaction of circadian and lunar rhythms that poses new challenges and offers new possibilities to explore the unknown molecular basis of lunar clockworks.

## Materials and methods

### Laboratory strains

Laboratory strains of Port-en-Bessin (Por, Normandie, 49° 21' 00'' N 0° 45' 10'' W) and St. Jean-de-Luz (Jean, Basque Coast, 43° 24' 50'' N 1° 39' 45'' W) were established from copulae that were caught in the field during September 2007 (Por) and October 2007 (Jean) respectively. The laboratory strains were reared according to Neumann [3]. Temperature in the climate chambers (Snijders Economic Premium) was at 20°C, relative humidity at 50% and the light dark cycle (LD) was 14:10 (lights on at 7 a.m.), with 5000 lux during the day and 45 minutes each of stepwise dusk and dawn (one step of 1250 lux every 15 min). Full moon was simulated with a standard incandescent torch light bulb (about 1 Lux) switched on all night for four successive nights every 30 days. All generations - parental strains, F1 and BC - were subject to two cycles of artificial moonlight pre-treatment before adult emergence started.

Since temperature and mechanical vibrations are known to act as zeitgeber for the lunar rhythm of *Clunio marinus *as well [25,26], we recorded both parameters in our climate chambers. Temperature was recorded with a digital minimum-maximum thermometer (TFA 30.1017.10) and did not vary by more than ± 0.6 °C across the whole experiment in each of the climate chambers. Vibration data were recorded with a vibration data logger (irDAN^®^vibra_c from ESYS) for 545 hours in one minute intervals and analyzed with Chronos-Fit software [47] for periodicity. The Lomp-Scargle periodogram (also called power spectrum) identified periods above threshold significance at 968.9, 272.5, 141.4, 108.5, 88.1, 71.7 and 61.2 hours. None of these is close to a tidal period (12.42 hours) or a multiple of it, precluding that the lunar rhythm could have been entrained by another zeitgeber than artificial moonlight. Furthermore, none of the significant periods is close to a 24 hour period plus the daily shift in diurnal emergence time observed in the BC, excluding that emergence was directly triggered by changes in vibration intensity.

The newly established strains were kept under laboratory conditions for one generation before starting the crosses.

### Crosses

In order to allow for genetic and molecular analysis, crosses were carried out as single pair matings. We first produced hybrids of the two strains in both directions (Por × Jean, Jean × Por) and then performed backcrosses to the Jean strain in both directions (F1 × Jean, Jean × F1). This is in contrast to Neumann [27] who backcrossed to the Por parental strain. Notably, the patterns of inheritance of diurnal emergence time were intermediate in both experiments. To be able to cross the strains and hybrids we had to synchronize their emergence peaks, both lunar and diurnal, by keeping them in separate climate chambers that were running at a phase-shifted light-dark cycle and with different days of artificial full moon.

F1 and BC were also raised under standard moonlight treatment. In the BC, two families with 57 or 20 individuals respectively were raised singly for future molecular analysis, these two families representing reciprocal crosses for both the F1 and the BC. A possible difference between these two backcross families in the lunar and diurnal emergence times was tested by comparing the phenotype distributions in a Kolmogorov-Smirnov test. We could not find significant differences in emergence times, neither lunar (major peak: p = 0.22; minor peak: p = 0.17) nor diurnal (lunar peaks summed up: p = 0.66). The other BC clutches were reared in mass cultures.

### Phenotype scoring

Lunar phenotypes were recorded for all, and diurnal phenotypes for most individuals. The lunar phenotypes (in units of days) are recorded as the day of the artificial moonlight cycle when the individual emerged, "day 1" being the first day with artificial moonlight in the laboratory cycle of 30 days. The diurnal phenotypes (in units of hours) were recorded in reference to the phase of the light-dark cycle in the respective climate chamber. Following Neumann [3], the middle of the dark phase was defined as "hour 0", thus making the middle of the light phase "hour 12". For the parental generation and the F1 generation, diurnal emergence times were recorded continuously while performing the crosses and later grouped into 30 minute intervals. For the backcross progeny, diurnal emergence times were recorded by catching all emerged midges in 30 minute intervals for the two single families, or in 1 hour intervals for the mass crosses. Each BC family emerged over two lunar cycles. As recording of diurnal phenotypes was not possible throughout the full emergence period of more than 2 months, diurnal emergence times are not available for all individuals, explaining the reduced numbers of individuals in analyses requiring diurnal emergence times. Data were summed over lunar cycles for analysis.

Both crossing and recording took place partly during the dark phase. During that time we worked under red light, using an custom-made hand-held LED lamp emitting a wavelength of 650 nm. While the midges are attracted to white light, they did not respond behaviourally to the red LED. As a control we also treated standard cultures with this lamp. We did not observe a shift in diurnal emergence times in these cultures, and so concluded that the red light presented in the first hours of the dark phase during our manipulations did not shift the circadian clock of the individuals in our experiment.

Due to malfunctioning thermostats, the parental strains and most of the F1 families were unintentionally raised at 14°C instead of 20°C, while the single reared backcross families were reared at 22°C instead of 20°C. We made no adjustment to the observed diurnal emergence phenotypes due to this, because previous experiments on *Clunio marinus *have shown that the diurnal time of emergence is not affected by temperature [48]. This is consistent with the well-known temperature compensation of endogenous circadian clocks [49,50] and the previously-established role of the circadian clock in directly controlling diurnal emergence in *Clunio *[3,48]. However, we did correct the lunar emergence phenotypes of the parental strains and the affected F1 progeny that were raised at 14°C by 1 day, because previous experiments on *Clunio marinus *have shown that reducing the temperature from 20°C to 14°C increases the pupal development interval by about 1 day [48]. The temperature increase from 20°C to 22°C is not expected to have a major effect [48]; no correction was applied in this case. Neumann has shown [3,51] that the developmental event controlled by the temperature-compensated lunar clock is not emergence from the pupa *per se*, but a certain stage in imaginal disk development in the early last larval instar. From this switching point larval develoment and thus timing of entry into the pupal stage are temperature compensated as well [51]. However, from the time of pupation to emergence of adults, pupal development is not temperature-compensated, and lower temperatures lead to later lunar emergence times. The correction we applied assumes no genetic component to the temperature-dependence of pupal development rate. The adjustment would not affect conclusions about the correlation coefficient of lunar and diurnal emergence times among the backcross progeny, which measures the pattern of variation about the means and was calculated and analysed independently for the single rearing (22°C) and the mass rearing (20°C). It could affect the goodness-of-fit tests for the number of loci controlling lunar emergence time, but should do so independently of the number of loci assumed. To test this, we repeated the test with uncorrected data; in this case, all genetic models are rejected at the 0.05 significance level, but the pattern of p values still has the same relation, thus the model which is the most likely remains the same, unaffected by the use of the uncorrected data.

### Statistical analysis

All graphs and statistics were calculated using the R [52] statistical programming environment.

To test for the number of genes involved in determining diurnal or lunar emergence time respectively, for the BC we calculated the expected distribution of phenotypes according to different numbers of genetic factors involved and tested the observed distribution of phenotypes against them. The expected distribution for one genetic factor was obtained by combining the observed distributions of the parents of the BC, i.e. the F1 and the Jean strain. For more genetic factors, we obtained the expected distribution by sampling 200,000 individuals from the observed distributions of the F1 and the Jean strain. For the process of sampling we divided the sample into the underlying genotypes according to Mendelian expectations, e.g. 1:2:1 for two genetic factors, 1:3:3:1 for three genetic factors and so on. The fraction of pure parental genotypes (F1, Jean) was sampled directly from the observed parental distributions. For mixed genotypes we sampled one phenotype each from the observed F1 and Jean distributions and averaged the values according to the ratio of genetic factors coming from F1 vs. Jean. The combined distribution of phenotypes of all genotypes was assumed to be the expected distribution for the respective number of genetic factors. The expected distribution was then scaled to the size of the original sample (67 for diurnal emergence times of the single families; 816 for diurnal emergence times in Neumann 1967; 487 for the lunar emergence times in the major peak of the BC) and compared to the observed distribution of the original sample in a Kolmogorov-Smirnov test, and in a G-test for independence referred to the Chi-square distribution with 18 degrees of freedom for the lunar data or 10 degrees of freedom for the circadian data respectively.

The correlation of diurnal and lunar emergence times was assessed by Pearson's product moment correlation. To test which degree of genetic linkage would be required to achieve a correlation of the given magnitude, we compiled matrices of all possible genotypes in the BC for all genetic models from 2-4 lunar and 2-4 circadian genes. Linkage models considered were of the following form: 0 linkages: all lunar genes unlinked to all circadian genes; 1 linkage: one lunar gene linked to one circadian gene; all others mutually unlinked; 2 linkages: same as 1 linkage but in addition a second lunar gene linked to a second circadian gene; etc. Depending on the pattern of genetic linkage assumed, we sampled genotypes for 62 individuals (number of individuals with known diurnal emergence time in the major BC peak of the single reared families) from the full matrix in case of freely recombining factors, or from the respective subsets of the matrix in the case of linkage. For these genotypes we then sampled phenotypes: For each gene coming from F1 or Jean strain we sampled a corresponding phenotype from the F1 or Jean distribution. Then these phenotypes were weighted according to the number of genes assumed to be involved in determining the respective trait, lunar or diurnal emergence time. The genes were assumed to have equal effects. The resulting lunar and diurnal phenotypes were grouped into classes according to those used in the observed BC and the correlation was assessed using Pearson's product moment correlation. We repeated this procedure 100.000 times for each genetic model. The fraction of p values smaller or equal to the p value of the observed BC distribution is the p value obtained, p_resamp_. If a model with a certain degree of linkage was already rejected, we did not test the genetic models that would allow for more independence of the traits, e.g. if a model with 3 circadian genes, 2 lunar genes and 2 linkages was rejected, we did not test the model with 4 circadian genes, 2 lunar genes and 2 linkages, as this model would necessarily imply a higher degree of independence of the two traits.

The timing traits of the laboratory strains relative to artificial moonlight were taken from [3,30,53]. If mean values and standard deviations were not given, we calculated them from the graphs.

The data on the local tidal regimes were taken from tide tables for the year 1979. As there is no notable difference between years, the choice of year does not matter. The tide tables, as well as all data and calculations based on them, are in Central European Time (CET). To obtain the interval between the light stimulus and the spring tides in the field we had to obtain a measure for these two events. To estimate the time of the moonlight stimulus, the date of all days with a low tide between 11 p.m. and 1 a.m. was noted. Usually this condition is fulfilled for 2 or 3 days in a row. There are two periods in a lunar cycle when low tides occur around midnight, but for every lunar cycle only the period during the moonlit quarters (i.e. the one closer to full moon) was considered, as only during this period will the moon be in the sky during the midnight low tide and can act as a zeitgeber. The spring tides occur around full moon and new moon, but the exact spring tide day varies by one or two days from place to place and across the year. Therefore we used the days of full moon and the days of new moon as a universal approximation of the spring tide days for all places. Finally, the time between the light stimuli and new moon or full moon respectively was averaged for all months of the year 1979. The time of low tide on full moon day or new moon day respectively was averaged for the whole year of 1979 as well.

## Authors' contributions

TSK conceived the study, designed the experiment, established the laboratory stocks, performed the crosses, analysed the data and wrote the manuscript. DN advised on laboratory culture, brought forward the interpretation of the data by continuous discussion and edited the manuscript. DGH advised on experiment design and data analysis and revised the manuscript. All authors read and approved the final manuscript.
